# A Town-Level Comprehensive Intervention Study to Reduce Salt Intake in China: Cluster Randomized Controlled Trial

**DOI:** 10.3390/nu14214698

**Published:** 2022-11-07

**Authors:** Min Liu, Jianwei Xu, Yuan Li, Feng J He, Puhong Zhang, Jing Song, Yifu Gao, Shichun Yan, Wei Yan, Donghui Jin, Xiaoyu Chang, Zhihua Xu, Yamin Bai, Ning Ji, Jing Wu

**Affiliations:** 1 National Center for Chronic and Non-Communicable Disease Control and Prevention, Chinese Center for Disease Control and Prevention (China CDC), Beijing 100050, China; 2 George Institute for Global Health, Peking University Health Science Center, Beijing 100600, China; 3 Wolfson Institute of Population Health, Barts and The London School of Medicine & Dentistry, Queen Mary University of London, London E1 4NS, UK; 4 Department for Chronic and Non-Communicable Disease Control and Prevention, Hebei Provincial Center for Disease Control and Prevention, Shijiazhuang 050024, China; 5 Department for Chronic and Non-Communicable Disease Control and Prevention, Heilongjiang Provincial Center for Disease Control and Prevention, Harbin 150030, China; 6 Department for Chronic and Non-Communicable Disease Control and Prevention, Jiangxi Provincial Center for Disease Control and Prevention, Nanchang 330029, China; 7 Department for Chronic and Non-Communicable Disease Control and Prevention, Hunan Provincial Center for Disease Control and Prevention, Changsha 410028, China; 8 Department for Chronic and Non-Communicable Disease Control and Prevention, Sichuan Provincial Center for Disease Control and Prevention, Chengdu 610044, China; 9 Department for Chronic and Non-Communicable Disease Control and Prevention, Qinghai Provincial Center for Disease Control and Prevention, Xining 810007, China

**Keywords:** twenty-four hour urinary sodium, salt reduction, randomized trial, sodium, China

## Abstract

We determined whether a town-level comprehensive intervention program could lower the salt intake of a population. The parallel, cluster randomized controlled trial was carried out between October 2018 and January 2020 in 48 towns from 12 counties across 6 provinces in China. All participants were asked to complete the 24 h urine collections, anthropometric measurements and questionnaires at the baseline and one-year post-intervention survey. A total of 2693 participants aged 18 to 75 years were recruited at the baseline. A total of 1347 individuals in 24 towns were allocated to the intervention group and the others were allocated to the control group. Valid information from 2335 respondents was collected in the follow-up survey. The 24-h urinary sodium excretion was 189.7 mmol/24 h for the intervention group and 196.1 mmol/24 h for the control group at baseline. At a one-year follow-up, the mean effect of salt intake did not show a significant change (*p* = 0.31) in the intervention group compared to the control group. However, the mean result of potassium excretion in the intervention group increased by 2.18 mmol/24 h (85.03 mg/24 h) (*p* = 0.004) and systolic blood pressure decreased by 2.95 mmHg (*p* < 0.001). The salt-related knowledge and attitude toward salt reduction improved significantly in the intervention group (*p* < 0.05). A longer period of intervention and follow-up assessment might be needed to evaluate the long-term effectiveness of the program on salt reduction.

## 1. Introduction

Studies have shown that excessive salt intake is associated with high blood pressure (BP), which is a major cause of cardiovascular disease (CVD), such as stroke and ischemic heart disease [1,2,3,4]. In China, the prevalence rate of hypertension in adults was 27.5% [5], and high blood pressure contributed to 2.33 million CVD deaths [6]. In order to reduce the harm caused by a high-salt diet, the World Health Organization (WHO) recommended that adults reduce their salt intake to lower than 5 g/d (87 mmol/d) [7]. However, the average salt intake of Chinese residents was 11.0 g per day per capita in 2020, more than twice the WHO’s recommended amount [8].

Salt reduction is one of the most cost-effective means of preventing cardiovascular disease and it was recommended as one of the best strategies to solve the global crisis in noncommunicable diseases [9]. Actions to reduce salt have been initiated in many countries [9,10,11,12]. For China, which is a country with the largest share of the world’s CVDs, more intervention studies on salt reduction are urgently needed. In order to achieve the goal of reducing salt intake, the Action on Salt China (ASC) program was launched in 2017 to implement a series of salt reduction projects targeting various settings (e.g., local restaurants, schools, hospitals and communities) and salt intake sources [13,14,15]. The Comprehensive Intervention Study (CIS) [16] was a community-based Randomized Controlled Trial (RCT) included in the ASC program [17,18,19] aimed at evaluating the acceptability, scalability and effectiveness of the comprehensive intervention and its components. In this study, we evaluate the one-year effectiveness of implementing the comprehensive salt reduction intervention, which provides evidence for a national promotion.

## 2. Materials and Methods

### 2.1. Design

A detailed description of the methods has been published elsewhere [16]. The study conducted a cluster RCT in 6 provinces, including Hebei, Jiangxi, Hunan, Sichuan, Heilongjiang and Qinghai, which accounted for the diversity in geographical distribution, economic level and dietary habits of the Chinese population. In total, 48 towns (clusters) were selected from 12 counties across 6 provinces. Two counties were chosen from each province. From each county, we selected four towns that were similar in development levels and population size and randomly divided them into the intervention group and control group. Twenty-eight eligible participants were selected from each village. In order to minimize contamination between the intervention group and control group, most towns were selected from rural and suburban zones where living environments are relatively isolated.

The baseline survey was carried out between October and December 2018. The one-year intervention procedures started after the baseline investigation was completed and the one-year follow-up evaluation was conducted between November 2019 and January 2020. The program was approved by the Queen Mary Research Ethics Committee (QMERC 2018/16) and the Institutional Review Board of the National Center for Chronic and Noncommunicable Disease Control and Prevention (201807). All participants signed written informed consent forms and they could withdraw from the study at any time.

### 2.2. Study Participants

Individuals aged 18 to 75 years and who had been local residents for over 6 months were eligible for inclusion in the outcome evaluation. Only one person could be selected from each family. We excluded women who were pregnant or in the lactation period, participants who were not suitable for 24 h urine collection, and patients with severe psychiatric and physical diseases.

### 2.3. Randomization and Masking

Towns (clusters) which were stratified by the province were randomized 1:1 to intervention and control groups using a computer-generated randomization sequence. Randomization took place after the baseline survey. The local participants and investigators were unaware of the assignment until the intervention began.

### 2.4. Data Collection

All of the participants were asked to complete the 24-h urine collections, anthropometric measurements and questionnaires at the baseline and evaluation survey. The 24-h urine samples were collected following the instruction from the trained research staff. The participants were asked to empty their bladders, note down the time at the start of collecting urine on the first day, collect all subsequent urine voids over the next 24-h period, and return the urine collection equipment with all of the 24-h urine samples on the second day with the time of the last urine collection recorded. If the participants reported that they forgot to gather or splashed more than 10% of their total amount of urine, or if blood, excrement, or other impurities had contaminated the urine sample, the 24-h urine samples needed to be collected again following the abovementioned procedures. The urine samples were tested for the concentrates of sodium, potassium and creatinine, and the 24-h urinary excretions of sodium, potassium and creatinine were calculated as the product of urine volume and concentrate.

The BP was measured three times by trained researchers using a validated automatic blood pressure monitor (OMRON: HEM-7120) following the standard protocol [16,17]. The mean of the last two BP measurements was included in the analysis. Height, weight, and outdoor temperature were recorded by qualified investigators using calibrated equipment. Body mass index (BMI) was calculated as weight (kg) divided by the square of height (m^2^).

Information on salt reduction knowledge, attitude and practice (KAP), and lifestyle factors (e.g., physical activity, and alcohol drinking) were collected in face-to-face questionnaires administered by trained researchers. KAP questions related to salt intake recommendations, low-sodium salt, the identification of sodium content on food labels, dietary tastes and consumption of processed foods. Physical activity was defined as participants self-reporting their participation in moderate physical activity for 30 min or more at least three times a week. Alcohol drinking status was classified as non-drinker (the respondents answered “no” to the question “do you drink alcohol”), occasional drinker (the respondents answered “sometimes”), and regular drinker (the respondents answered “always” or “addicted”). All of the baseline and 12-month data were managed within a mobile electronic data collection system [20].

### 2.5. Intervention

The intervention package was designed by the CIS national project office, and implemented by the local Centers for Disease Control and Prevention (CDC). The researchers trained local health educators in a three-day workshop. Detailed intervention workbooks and lecture courses were offered. The salt reduction education included three parts: salt and health; salt reduction target; and how to reduce salt intake and use the salt substitute. The salt substitute courses introduced the benefits of low-sodium salt and how to choose low-sodium salt. The details of the intervention procedures and resources have been described before [16].

To achieve better intervention effects, a multifaceted comprehensive salt reduction strategy of proposed based on the existing evidence from other countries [1,3,11,21], and was implemented by the county, township and village local governments, respectively. Other major stakeholders, such as hospitals, schools, restaurants, and publicity departments were also engaged in the development of the intervention.

#### 2.5.1. Salt Reduction Campaign

Various kinds of salt reduction-related activities were carried out in the intervention group. Community residents were supplied with educational materials including posters, brochures, leaflets and signs. Salt reduction videos were broadcasted in public places such as parks and buses. Salt reduction publicity was carried out on at least two publicity days or important holidays every year, such as world salt reduction week and national hypertension day. Mass culture and publicity activities related to “salt and health” were organized at least once a year, such as knowledge competitions, family healthy cooking competitions, or other activities, to create a better salt reduction environment. In order to better cover young and middle-aged people, we also promoted salt and health knowledge and skills through social media, such as WeChat public accounts.

#### 2.5.2. Salt Reduction Interventions in Primary Health Centers

Besides public education, CIS emphasized the proactive role of primary health centers in the implementation of the salt reduction intervention. The county-level CDC integrated the training with the National Basic Public Health Service and conducted training at least twice a year for all primary health care providers in the intervention group. Each primary health center held at least two salt reduction lectures and activities every year using standardized teaching materials. Primary health care providers would impart knowledge and tips on salt reduction during routine outpatient visits to improve patients’ KAP toward salt reduction, thereby reducing the burden of hypertension and CVDs attributed to excessive salt intake. In order to achieve a good effect in rural areas, salt reduction knowledge was also publicized in the form of broadcasting among villagers. Salt-restricting spoons (2 g per spoon) for measuring salt during cooking were distributed to the family chef.

#### 2.5.3. Salt Reduction Interventions in Schools

At all of the schools in the intervention group, publicity posters were put up on bulletin boards or school canteens. Health education activities related to salt reduction were carried out at least once a year in teacher training and school–parent meetings. Salt reduction education courses were carried out to promote the harm of a high-salt diet to students. Public activities, such as making salt-related handwritten art and other patterns were conducted in schools.

#### 2.5.4. Salt Reduction Interventions in Restaurants

Information about salt reduction, health and salt was displayed through posters, videos and table stickers in restaurants to create an environment conducive to salt reduction. The chef and waiters in the restaurants were offered standardized training at least four times a year on how to reduce salt usage during cooking and how to guide customers to choose lower-salt dishes. Lower-salt dishes were marked on the menu to make it easier for the customers to choose.

Participants in the control group carried on with the usual health education (Health Action for All, Basic National Public Health Service, and so on) and no additional interventions on salt reduction were conducted during the intervention period.

### 2.6. Outcomes

The primary outcome included the difference in the change in 24-h urinary sodium, urinary potassium, the sodium–potassium ratio and BP from the baseline to the end of the trial between the intervention group and the control group. Secondary outcomes were the differences in the changes from KAPs on salt reduction from the baseline to the end of the trial between the intervention group and the control group.

### 2.7. Data Analysis

The effect of the town-level comprehensive intervention on the outcomes was analyzed using a general linear mixed model with a random intercept assessing the 3-level clusters (individual-level data were nested at the village level, and the village-level data were nested at the county level). The independent variables included group (control and intervention), time (baseline and 12-month), and time × group interaction. The time × group interaction term means the difference in the change in outcome measurement over the 12 months from the baseline between the intervention group and the control group. Stratification variables at randomization (towns) and potential confounding variables including age group (<40 = 1, 40~60 = 2, ≥60 = 3), sex (male = 0, female = 1), education level (primary education or less = 1, secondary school = 2, high school = 3, university or college = 4), BMI, outdoor temperature, physical activity and alcohol drinking status were adjusted in the general linear mixed models.

The Intent-To-Treat (ITT) analyses were used, but possibly incomplete 24-h urine collections were excluded in the primary analyses of urinary outcome measures. We defined the possibly incomplete 24-h urine samples as urine volume <500 mL/24-h, or creatinine <6.0 mmol/24-h in men or <4.0 mmol/24-h in women. Urine samples with collection times <20 h or >28 h were also excluded. If the 24-h urine samples were defined as incomplete at either baseline or 12 months, we used only the complete samples. In total, we excluded 409 urine collections from 5386 for the primary analyses of urinary outcome measures. To examine the robustness of the conclusions from the primary analysis, we also carried out two sensitivity analyses of urinary outcome measures: (1) in all the participants who attended the two surveys, and (2) in participants who completed both baseline and 12-month assessments (named as completers).

We used SAS (version 9.4) for data analysis. Continuous variables were described as means and standard deviations (SDs) and presented as mean estimates and 95% confidence intervals (CI) in the inferential analyses. Categorical variables were described as the frequencies and percentages, odds ratios (ORs) and 95% CI in the inferential analyses. The *t*-test and chi-square test were used to describe the difference in the characteristics of the participants between the intervention group and the control group. All analyses were two-sided, and *p* < 0.05 was considered significant.

## 3. Results

### 3.1. Baseline Characteristics of Participants

**Figure 1:**
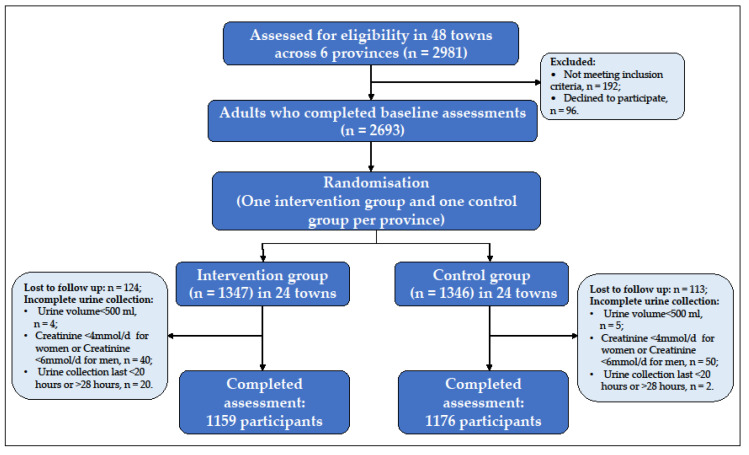
Flowchart of participants in the baseline and follow-up surveys.

A total of 2981 adult participants were recruited in 48 towns (8 towns in each province, 2 communities in each town) from six provinces. 288 respondents were excluded because they did not meet the inclusion criteria (*n* = 192) or refused to participate (*n* = 96). 2693 participants completed a baseline assessment. After randomization, 1347 individuals (from 24 towns) were allocated to the intervention group and 1346 persons (from 24 towns) were allocated to the control group. During the trial, 237 (8.8%) persons were lost before the 12-month follow-up evaluation, due to moving to other places, a long time out of work, or being unable to attend the follow-up assessment. After excluding 121 incomplete urine samples in the evaluation survey, the sample size was reduced to 2335 participants. Figure 1 shows the baseline and follow-up numbers for the intervention and control groups.

Table 1 shows the baseline characteristics of the participants in the control and intervention groups. The mean age of the 2693 participants was 48.0 years, and 49.5% of them were men. The mean BMI was 24.7 kg/m^2^. The two groups were well balanced in most parameters except age, education status, self-reported hypertension and blood pressure treatment in the self-reported hypertensive. The age, self-reported hypertension and blood pressure treatment were higher in the intervention group and the control group had a higher level of education.

### 3.2. Primary Outcome

Table 2 shows the covariates-adjusted mixed linear model result of the urinary outcomes and blood pressure. The mean baseline 24-h urinary sodium excretion was 196.1 mmol/24 h (equivalent to 11.5 g/d of salt) in the control group and 189.7 mmol/24 h (equivalent to 11.1 g/d of salt) in the intervention group. After one-year follow-up, sodium excretion does not significantly change in either the intervention group or control group (change from baseline in the intervention group: −0.27 mmol/24 h, *p* = 0.91; change from baseline in the control group: −4.00 mmol/24 h, *p* = 0.12). Comparing the intervention with the control group, the mean effect on salt intake did not show a significant change (*p* = 0.31).

The 24-h urinary potassium excretion decreased in the control group after the one-year follow-up while no changes were observed in the intervention group (change from baseline in the intervention group: 0.53 mmol/24 h, *p* = 0.32; change from baseline in the control group: −1.65 mmol/24 h, *p* = 0.002). The comparison of the change in urinary potassium excretion between the intervention group and the control group shows a significant intervention effect on increasing the urinary potassium (2.18 mmol/24 h, equivalent to 85.03 mg/24 h) (*p* = 0.004).

After adjusting for the stratification variables at randomization and the confounding factors, the systolic blood pressure was decreased in the intervention group from baseline, but does not change in the control group (change from baseline in the intervention group: −2.24 mmHg, *p* < 0.001; change from baseline in the control group: 0.71 mmHg, *p* = 0.13), and the intervention was estimated to have lowered the systolic blood pressure by −2.95 mmHg (95%CI: −4.08 mmHg to −1.83 mmHg, *p* < 0.001) after the one-year follow-up. Comparing the 12-month changes in other outcomes in the intervention group with those estimated in the control group, there was no observed intervention effect on the diastolic blood pressure and sodium-to-potassium ratio after the intervention (*p* > 0.05), but a significant effect in increasing the 24-h urine volume (121.57 mL/24 h, *p* < 0.001).

Supplementary Table S1 shows the results of sensitivity analyses. The results were similar to those from the primary analyses. The mean effect on 24-h urinary sodium excretion was unchanged when the data included possible incomplete 24-h urine collections or only included the participants who completed the baseline and end trial assessment with complete 24-h urine collections. Supplementary Table S2 shows the subgroup results. The 24-h urinary sodium excretion does not significantly change in any of the subgroups between the intervention and control groups.

### 3.3. Secondary Outcomes

Table 3 shows the results of the knowledge, attitude and behaviors of salt reduction in the baseline and 12-month survey. Comparing the intervention with the control group, there is a significant intervention effect on increasing the proportion of participants with the knowledge of salt intake recommended by Chinese nutrition guidelines after the one-year follow-up (OR = 9.43 (7.28–12.21), *p* < 0.001), having heard about the low-sodium salt substitute (OR = 2.20 (1.73–2.80), *p* < 0.001), having the ability to identify the salt content on nutrition labels (OR = 3.50 (2.76–4.43), *p* < 0.001) and the willingness to choose a low-sodium diet (OR = 1.99 (1.48–2.66), *p* < 0.001). No significant difference was seen in the proportion of participants who prefer a less salty taste and who use a low-sodium salt substitute. After the intervention, the frequency of eating processed foods once per week or less increased in the intervention group (OR = 1.34 (1.14–1.58), *p* < 0.001)), but there was no significant change in the control group (OR = 1.10 (0.94–1.29), *p* = 0.22). The mean effect of eating processed food frequency does not significantly change when comparing the intervention group with the control group (OR = 1.22 (0.97–1.52), *p* = 0.09).

## 4. Discussion

The study was a large-scale town-level comprehensive intervention study designed to reduce salt intake in China. Although the 24-h urinary sodium excretion did not change with the one-year comprehensive intervention, the findings showed that the intervention did increase the 24-h potassium excretion, and significantly reduced systolic blood pressure. Sodium-related knowledge and attitude improved significantly following the comprehensive intervention.

We used 24-h urine collection which was the most accurate method to estimate the salt intake levels in the community adults [22]. The baseline data showed that the 24-h sodium excretion was excessively high (>4300 mg/d sodium or >11.0 g/d salt), whereas the potassium excretion was insufficiently low (<1600 mg/d). These findings were consistent with the result of the latest meta-analysis which demonstrated that the published 24-h urinary sodium and potassium levels in China over the past 40 years were 11.06 g/d and 1.42 g/d, respectively [23], highlighting the importance of reducing the salt intake and increasing the potassium intake in the Chinese population to reduce the disease burden attributed to the high-sodium and low-potassium diet. To tackle this issue, the Chinese government set a target of a 20% reduction in salt consumption in adults by 2030 as the key component of “Health China 2030” [24]. Our study was set up to develop an evidence-based and comprehensive salt reduction intervention to help achieve China’s salt reduction goal.

Various regional multifaceted salt reduction programs have been undertaken in China, such as SMASH (Shandong Ministry of Health Action on Salt Reduction and Hypertension) and the Resolve to Save Lives project [25]. Different from the findings of our study, the SMASH program which was a five-year intervention to reduce sodium consumption in Shandong province indicated that the government-led and population-based intervention led to a decline in dietary sodium intake, as well as in blood pressure [25]. However, there were some other previous studies on comprehensive salt reduction interventions reporting non-significant intervention effects on the population’s salt intake, which were similar to our results [26,27,28]. The conflicting in-study findings might be related to several reasons. First, the duration of the salt reduction intervention (e.g., one year) might only be enough to improve people’s knowledge and understanding of salt intake, but not sufficient to modify the dietary behavior and taste for foods in the community setting. Some of the studies that have successfully reduced salt intake in community populations lasted for more than three years [25,29,30]. This is partially supported by our results of the KAP outcomes that the one-year intervention managed to improve the knowledge and attitudes toward salt reduction, but no intervention effects were observed for either the frequency of having salty processed foods or the use of low-sodium salt substitutes. Compared with knowledge and attitude, behavior change is complex, long-term and slow [31,32]. The one-year intervention time was limited, and it was difficult for the intervention population to transform the knowledge and attitude of salt reduction into behaviors. Secondly, our study showed that the urine sodium excretion in both the intervention group and the control group decreased after one year, which might be partially explained by the ongoing salt reduction initiatives nationwide. For example, the Chinese government launched a nationwide campaign called “Healthy Lifestyle for All”, including calling for the reduction of the population’s salt intake [33]. Thirdly, there were differences in age, education level, prevalence and the treatment of hypertension, especially in the 24-h urinary sodium between the intervention group and the control group at baseline. It might take a long time for the intervention group with an older age group, lower education level, a higher prevalence of hypertension and a lower salt intake to obtain positive effects.

In addition to the 24-h urine sodium excretion, our study also showed an increase in urine potassium excretion and a fall in systolic blood pressure after the intervention. The comparison results within each group showed that the urine potassium excretion decreased in the control group and increased in the intervention group after the intervention, but the change in the intervention group was not statistically significant. This phenomenon may be related to various factors. Firstly, the time of follow-up surveys between the two groups was inconsistent. The time of the control group was one month later than the intervention group, which was closer to winter when the intake of fruits and vegetables was less. Many studies have shown that an adequate intake of vegetables and fruits could increase potassium excretion and help lower blood pressure [34,35]. Secondly, maybe due to the promotion of a healthy diet (including eating more vegetables and fruits and using a salt substitute), the intervention group had a slight increase in urinary potassium. Because of the decrease in urinary sodium and increase in urinary potassium, there was a decrease in systolic blood pressure in the intervention group compared with the control group. After the intervention, there was a significant difference in urine volume between the two groups. This may be related to the fact that the health education course included an introduction to the recommended daily water intake per person (no less than 1500 mL).

Strengths of our study include the large sample size from six provinces, cluster randomized controlled trial design, and comprehensive salt reduction interventions. Another strength is the collection of 24-h urine samples, which was widely acknowledged as the “gold standard” for measuring individual sodium intake [22]. In addition, careful efforts were made to standardize the collection of 24-h urine under the support of a bespoke electronic data capture system.

This study also had some limitations. First, a single year of intervention is not enough to modify the dietary behaviors in the community setting. Our research will continue to collect follow-up information for the second and third years after the completion of the intervention. Second, only one 24-h urine sample was collected from all participants, which cannot reflect the day-to-day variation in sodium and potassium excretion. This may have reduced the stability and reliability of the result. Third, data on the food consumed and the actual use of low-sodium salt substitutes was not collected, thus it would be difficult to understand the reasons for the changes in potassium levels in the study.

## 5. Conclusions

Although the 24-h urinary sodium excretion level did not change after one year of salt reduction intervention, the population potassium intake was observed to have increased and the systolic blood pressure had significantly decreased. Furthermore, people’s knowledge and attitudes significantly improved over the one-year intervention. A longer period of intervention and follow-up assessment might be needed to evaluate the long-term effectiveness of the intervention program on salt reduction in the communities. In order to achieve the “Health China 2030” target, effective public health policies and targeted interventions for salt reduction are urgently needed.

## Figures and Tables

**Table 1:** Participants’ baseline characteristics.

Characteristics	Control (*n* = 1347)	Intervention (*n* = 1346)	*p*-Value
Age (year), Mean (SD)	47.2 (13.0)	48.8 (12.6)	<0.001
Men, *n* (%)	667 (49.5)	667 (49.6)	0.98
Weight (kg), Mean (SD)	63.3 (12.1)	63.1 (11.4)	0.72
BMI (kg/m^2^), Mean (SD)	24.7 (3.7)	24.8 (3.5)	0.39
Physical activity: active *n* (%)	567 (42.1)	519 (38.6)	0.06
Education status (*n*, %)			
Primary education or less	540 (40.1)	596 (44.3)	0.02
Secondary school	536 (39.8)	503 (37.4)	
High school	159 (11.8)	161 (12.0)	
University or college	112 (8.3)	86 (6.4)	
Alcohol drinkers ^a^ (*n*, %)			0.82
Non-drinkers	792 (58.8)	800 (59.4)	
Occasional drinkers	426 (31.7)	415 (30.8)	
Regular drinkers	128 (9.5)	131 (9.7)	
Urine creatinine (mmol/24 h)	10.7 (3.3)	10.5 (3.5)	0.13
Self-reported hypertension ^b^ (*n*, %)	233 (17.4)	300 (22.3)	<0.001
BP treatment in self-reported hypertensives, (*n*, %) ^c^	157 (66.8)	247 (82.3)	<0.001

^a^ Number of participants with missing value = 1; ^b^ Number of participants with missing values = 5; ^c^ Number of self-reported hypertensive patients = 533.

**Table 2:** Results for 24-h urinary sodium excretion, other urinary measurements, and blood pressure from the covariates-adjusted mixed linear model.

	Control			Intervention			Adjust Difference * (Intervention vs. Control) (95% CI)	*p*-Value
	Baseline N, Mean (SD)	12-Month N, Mean (SD)	Model-Based Change from Baseline * (95% CI)	Baseline N, Mean (SD)	12-Month N, Mean (SD)	Model-Based Change from Baseline * (95% CI)		
Salt intake (g/d)	1327	1159	−0.23	1315	1176	−0.02	0.22	0.31
	11.5 (4.8)	11.3 (4.7)	(−0.53 to 0.06)	11.1 (4.5)	11.1 (4.8)	(−0.31 to 0.27)	(−0.20 to 0.64)	
Urinary sodium (mmol/24 h)	1327	1159	−4.00	1315	1176	−0.27	3.72	0.31
	196.1 (81.3)	192.6 (80.9)	(−9.06 to 1.07)	189.7 (77.0)	189.1 (82.4)	(−5.33 to 4.78)	(−3.43 to 10.87)	
Urinary sodium (mg/24 h)	1327	1159	−91.99	1315	1176 4349.8	−6.40	85.58	0.31
	4510.6 (187.0)	4430.9 (1860.8)	(−208.47 to 24.50)	4363.0 (1770.9)	(1894.2)	(−122.63 to 109.83)	(−78.82 to 249.99)	
Urinary potassium (mmol/24 h)	1327	1159	−1.65	1315	1176	0.53	2.18	0.004
	40.6 (16.8)	39.0 (15.1)	(−2.70 to −0.61)	39.6 (16.3)	40.3 (16.3)	(−0.52 to 1.57)	(0.70 to 3.66)	
Urinary potassium (mg/24 h)	1327	1159 1519.9	−64.45	1315	1176	20.58	85.03	0.004
	1584.6 (654.0)	(589.4)	(−105.30 to −23.60)	1546.2 (637.0)	1573.4 (634.6)	(−20.18 to 61.34)	(27.38 to 142.68)	
sodium-to-potassium ratio	1327	1159	0.09	1315	1176	−0.07	−0.17	0.11
	5.2 (2.3)	5.3 (2.3)	(−0.05 to 0.24)	5.1 (2.1)	5.0 (2.2)	(−0.22 to 0.07)	(−0.37 to−0.04)	
Systolic blood pressure (mm Hg)	1347	1242	0.71	1346	1231	−2.24	−2.95	<0.001
	125.6 (19.3)	128.0 (20.0)	(−0.21 to 1.64)	127.2 (19.3)	126.8 (18.8)	(−3.21 to −1.27)	(−4.08 to −1.83)	
Diastolic blood pressure (mm Hg)	1347	1242	−0.89	1346	1231	−1.34	−0.45	0.22
	79.0 (11.8)	79.1 (12.3)	(−1.49 to −0.29)	80.1 (11.4)	80.0 (11.5)	(−1.97 to −0.71)	(−1.17 to 0.27)	
Urinary creatinine (mmol/24 h)	1327	1159	−0.57	1315	1176	−0.27	0.30	0.01
	10.8 (3.2)	10.1 (3.2)	(−0.74 to −0.41)	10.6 (3.4)	10.3 (3.4)	(−0.44 to −0.11)	(0.06 to 0.53)	
Urine volume (mL/24 h)	1327	1159	−43.01	1315	1176	78.55	121.57	<0.001
	1608.3 (643.0)	1565.7 (631.1)	(−79.71 to −6.32)	1623.2 (652.4)	1711.9 (699.2)	(41.95 to 115.16)	(66.79 to 173.34)	

* Model-based change for urinary outcomes were adjusted for age categories, sex, education level and BMI at baseline and follow-up. Model-based changes for blood pressure were further adjusted for outdoor temperature at baseline and follow-up, physical activity and alcohol drinker status.

**Table 3:** Results for knowledge, attitude and behaviors of salt reduction in baseline and 12-month surveys from the covariates-adjusted mixed linear model.

	Control			Intervention			Intervention Effect * (Intervention Group vs. Control Group, OR, 95% CI)	*p*-Value
	Baseline *n* (%)	12-Month *n* (%)	Model-Based Change (OR, 95% CI)	Baseline *n* (%)	12-Month *n* (%)	Model-Based Change (OR, 95% CI)		
Knowledge								
Knowledge of the salt intake recommended by the Chinese nutrition guidelines (6 g/d)	330	375	1.38	233	889	12.99	9.43 (7.28,12.21)	<0.001
	(24.5)	(30.4)	(1.16, 1.64)	(17.3)	(71.6)	(10.73, 15.72)		
Having heard about a low-sodium salt substitute	342	458	1.81	371	725	3.99	2.20 (1.73, 2.80)	<0.001
	(25.4)	(37.1)	(1.52, 2.15)	(27.6)	(58.4)	(3.37, 4.72)		
Having the ability to identify salt content on a food label	577	505	0.97	504	784	3.40	3.50 (2.76, 4.43)	<0.001
	(42.8)	(40.9)	(0.83, 1.15)	(37.4)	(63.2)	(2.87, 4.03)		
Attitude								
Willingness to choose a low-sodium diet	1075	991	1.02	1067	1099	2.03	1.99 (1.48, 2.66)	<0.001
	(79.8)	(80.3)	(0.84, 1.24)	(79.3)	(88.6)	(1.63, 2.53)		
Preferring a less salty taste	380	372	1.08	366	369	1.12	1.04 (0.81, 1.32)	0.77
	(28.2)	(30.2)	(0.91, 1.28)	(27.2)	(29.7)	(0.94, 1.33)		
Behaviors								
Using a low-sodium salt substitute ^a^	109	133	0.91	130	239	1.01	1.11 (0.74, 1.67)	0.62
	(31.9)	(29.0)	(0.67, 1.24)	(35.0)	(33.0)	(0.77, 1.32)		
Eating processed food once per week or less	746	714	1.10	788	814	1.34	1.22 (0.97, 1.52)	0.09
	(55.4)	(57.9)	(0.94, 1.29)	(58.5)	(65.6)	(1.14, 1.58)		

^a^ Questions about using low-sodium salt substitutes were surveyed among people who have heard about low-sodium salt substitutes; * Model-based change for urinary outcomes were adjusted for age categories, sex, education level, and BMI at baseline and follow-up.

## Data Availability

Data are available upon request.

## Supplementary Materials

The following supporting information can be downloaded at: https://www.mdpi.com/article/10.3390/nu14214698/s1, Table S1: Sensitivity analysis for 24-h urinary measurements from the covariates-adjusted mixed linear model in the baseline and 12-month surveys; Table S2: Salt intake (g/day) as measured by the 24-h urinary sodium excretion from a subgroup in the baseline and 12-month surveys.
