# Supplementary material for: A Town-Level Comprehensive Intervention Study to Reduce Salt Intake in China: Cluster Randomized Controlled Trial
Source: Nutrients. 2022 Nov 7;14(21):4698. doi: 10.3390/nu14214698 (PMC9654622; doi:10.3390/nu14214698)
Supplement: Supplementary file 1 [file nutrients-14-04698-s001.zip › nutrients-1923755-supplementary.pdf]

Table S1. Sensitivity analysis for 24-hour urinary measurements from covariates-adjusted mixed linear model in baseline and 12-month survey

Primary and secondary outcomes	Control		Intervention		Difference of change* (intervention *vs* control) (95% CI)	*p*-value
	No. in baseline/12 months	Model-based change from baseline* (95% CI)	No. in baseline/12 months	Model-based change from baseline* (95% CI)		
**Including possible incomplete 24h urine**						
Urinary sodium (mmol/24h)	1347/1223	-6.26(-11.26 to -1.26)	1346/1233	-2.80(-7.80 to 2.19)	3.45(--3.61 to 10.52)	0.33
Urinary potassium (mmol/24h)	1347/1223	-2.11(-3.15 to -1.07)	1346/1233	0.02(-1.02 to 1.06)	2.13(0.66 to 3.60)	0.005
sodium-to-potassium ratio	1347/1223	0.09(-0.05 to 0.23)	1346/1233	-0.05(-0.19 to 0.09)	-0.14(-0.35 to 0.06)	0.16
**Completers**						
Urinary sodium (mmol/24h)	1144/1144	-4.19(-9.41 to 1.03)	1153/1153	-0.29(-5.49 to 4.91)	3.90(-3.47 to 11.27)	0.30
Urinary potassium (mmol/24h)	1144/1144	-1.63(-2.71 to -0.55)	1153/1153	0.48(-0.59 to 1.56)	2.12(0.60 to 3.64)	0.006
sodium-to-potassium ratio	1144/1144	0.09(-0.06 to 0.24)	1153/1153	-0.06(-0.21 to 0.09)	0.15(-0.36 to 0.06)	0.16

* Model-based change for urinary outcomes are adjusted for age categories, sex, BMI at baseline and follow-up.

Table S2. Salt intake (g/day) as measured by 24-hour urinary sodium excretion by subgroup in baseline and 12-month survey

	Control			Intervention			Adjust difference * (intervention vs control) (95% CI)	*p*-value
	Baseline *n*, Mean (SD)	12-Month *n*, Mean (SD)	Model-based change from baseline* (95% CI)	Baseline *n*, Mean (SD)	12-Month *n*, Mean (SD)	Model-based change from baseline* (95% CI)		
**Sex**								
Male	656 12.0(5.1)	562 11.9((5.1)	-0.15(-0.60, 0.30)	646 11.7(4.7)	566 11.8(5.3)	0.15(-0.30, 0.60)	0.30(-0.33, 0.93)	0.35
Female	671 11.0(4.4)	597 10.7(4.2)	-0.32(-0.71, 0.07)	669 10.5(4.2)	610 10.4(4.2)	-0.17(-0.56, 0.21)	0.15(-0.40, 0.70)	0.60
**Age**								
<40	405 11.8(4.7)	323 11.7(4.7)	-0.21(-0.80, 0.38)	343 11.2(4.8)	263 11.4(5.1)	0.11(-0.53, 0.76)	0.32(0.56, 1.20)	0.47
40-<60	547 11.6(4.8)	582 11.3(4.8)	-0.35(-0.77, 0.07)	700 11.3(4.4)	642 11.3(4.9)	-0.02(-0.42, 0.39)	0.33(-0.24, 0.91)	0.26
≥60	265 10.7(4.6)	254 10.6(4.5)	-0.08(-0.68, 0.50)	272 10.4(4.3)	271 10.2(4.2)	-0.16(-0.74, 0.42)	-0.08(-0.90, 0.75)	0.86
**Education level**								
Primary school or lower	534 11.9(4.9)	462 11.6(5.0)	-0.36(-0.84, 0.10)	583 11.0(4.6)	507 10.9 (4.4)	-0.08(-0.53, 0.37)	0.28(-0.37, 0.94)	0.39
Secondary school	527 11.5(4.9)	462 11.3(4.7)	-0.04(-0.53, 0.44)	490 11.4(4.6)	438 11.5(5.2)	0.01(-0.49, 0.52)	0.06(-0.65, 0.76)	0.87
High school	154 11.0(4.4)	141 10.7(4.5)	-0.40(-1.27, 0.47)	157 11.1(4.1)	157 11.4(5.4)	0.25(-0.59, 1.10)	0.65(-0.55, 1.87)	0.29
University or college	112 10.0(3.2)	94 10.1(3.5)	-0.04(-0.87, 0.79)	85 10.1(4.3)	74 9.3(3.4)	-0.73(-1.68, 0.21)	-0.69(-1.95,0.56)	0.27

SD=standard deviation.

* Model-based change for urinary outcomes are adjusted for age categories, sex, BMI at baseline and follow-up.
